# From the bench to the bedside: Emerging science in Parkinson’s disease, cholesterol metabolism, and neuroprotection

**DOI:** 10.4103/2152-7806.68339

**Published:** 2010-08-10

**Authors:** Jason S. Hauptman

**Affiliations:** Intellectual and Developmental Disabilities Center and Department of Neurosurgery, Geffen School of Medicine at UCLA, Los Angeles, CA

## IN THIS UPDATE

- Parkinsonian motor symptoms are recapitulated *in vivo* by selectively activating the indirect and direct basal ganglia pathways.
- Cholesterol metabolism by the liver is shown to be regulated by brain circuits.
- A new chemical has been described that enhances neurogenesis, protects neurons from cell death, and reduces aging-related cognitive decline.

## MANIPULATION OF BASAL GANGLIA CIRCUITS *IN VIVO*: NEW INSIGHTS INTO PARKINSONISM[1]

### Key points

1. Channelrhodopsin-2 (ChR2) is a protein that allows for light-activation of the neurons that express it, and can be targeted to very specific neuronal populations of interest. In this article, the authors use ChR2 to selectively activate either D1- or D2-positive medium spiny neurons (MSNs) in the striatum.
2. In the striatum, D1-positive MSNs are thought to mediate the direct pathway, whereas D2-positive MSNs mediate the indirect pathway. Activation of the direct pathway unilaterally causes contralateral rotation, whereas the indirect pathway causes ipsilateral rotation. Bilateral activation of the direct pathway causes increased ambulation and decreased freezing, whereas bilateral activation of the indirect pathway causes bradykinesia, decreased locomotion, and freezing.
3. Bilateral activation of the direct pathway rescues symptoms of Parkinson’s disease.

Classical neuroanatomy teaches that the basal ganglia have two competing circuits that direct motor planning and action: the direct pathway that facilitates movement and the indirect pathway that inhibits it. Although this theory of basal ganglia function is based on the anatomical connections within the striatum, it has yet to be proven empirically. Until now, that is, in this study by Kravitz *et al*., researchers expressed the membrane protein ChR2 in MSNs of the basal ganglia that have either D1 or D2 dopamine receptors (in some animals ChR2 was expressed only in the D1-positive MSNs, whereas in others ChR2 was expressed only in D2-positive MSNs). The D1 and D2 dopamine receptors are metabotropic, meaning they trigger a cascade of protein activation or inactivation leading to the generation of intracellular signals. In the case of D1 receptors, activation leads to generation of the intracellular messenger cyclic adenosine mono-phosphate (cAMP). Activation of D2 receptors, on the other hand, leads to inhibition of cAMP formation. These opposing effects of D1 and D2 receptors are due to actions on different G protein subunits at the cell membrane. ChR2 is a unique protein that causes neurons to depolarize (i.e., fire an action potential) when exposed to particular wavelengths of light. According to theories of basal ganglia function, activation of D1-expressing MSNs is unique to the direct pathway, whereas activation of D2-expressing MSNs occurs in the indirect pathway. To prove this, researchers selectively activated these MSNs using the ChR2 proteins uniquely inserted into the membranes of either the D1 or D2 MSNs.

After confirming appropriate targeting of ChR2 to the MSNs and making sure that ChR2 did not change the physiological properties of the MSNs, the researchers inserted fiber-optic probes into the basal ganglia and demonstrated that by shining light they were able to modulate the firing patterns of the MSNs that expressed ChR2. Then, researchers placed recording electrodes in the substantia nigra pars reticulate (SNr), an output nucleus of the basal ganglia. When the researchers activated the D1-positive (direct pathway) MSNs, the firing rates of neurons in the SNr decreased. On the other hand, when the D2-positive neurons were activated, firing rates of neurons in the SNr increased. This is consistent with classical notions regarding the direct and indirect pathways.

In the final set of experiments, the researchers placed fiber-optic probes into the bilateral dorsomedial striatum to see how manipulating the indirect and direct pathways *in vivo* affects motor behavior. When the D1-positive (direct pathway) MSNs were activated unilaterally, the mice rotated towards the opposite direction. When the D2-positive (indirect pathway) MSNs were activated unilaterally, mice rotated towards the side being illuminated. Again, this is consistent with classical principles of basal ganglia action. How about when both sides were activated? When the D1-positive (direct pathway) neurons were activated bilaterally, mice spent more time ambulating at consistent speeds, less time freezing, and more time engaged in fine movements. However, when D2-positive (indirect pathway) neurons were bilaterally illuminated, mice suffered from bradykinesia, less locomotor initiation, slower ambulatory speeds, and increased freezing—thus, more Parkinsonian symptoms. This is consistent with the notion that symptoms of Parkinson’s disease are due to increased indirect pathway activity and less direct pathway activity. Need more proof? In the final experiment, investigators activated the D1-positive neurons in animals that received 6-hydroxydopamine lesions, a model of Parkinson’s disease. As expected, direct pathway activation completely alleviated the animals of their Parkinsonian symptoms—bradykinesia, freezing, and decreased locomotion. Deep brain stimulation, meet your match.

## CHOLESTEROL METABOLISM: IT IS NOT JUST THE GUT ANYMORE[2]

### Key points

1. The gut-brain axis plays an important role in the regulation of metabolism and feeding behavior. This includes signaling by hormones such as ghrelin, glucagon-like peptide-1 (GLP-1), and melanocortin. In general, ghrelin antagonizes melanocortin signaling and results in obesity, hyperphagia, and increases in cholesterol levels. GLP-1 and melanocortin, on the other hand, promote reductions in body mass, appetite, and cholesterol levels.
2. Modulation of melanocortin signaling affects high-density lipoprotein (HDL) cholesterol levels primarily by altering the hepatic reuptake of HDL (by changing the expression levels of Scarb1). This circuit also changes hepatic biosynthesis of cholesterol, but this seems to be less important mechanistically.
3. Chronic obesity and consumption of a high-fat diet results in decreases in melanocortin signaling but also hypersensitivity of melanocortin receptors, suggesting that restoration of melanocortin signaling may result in beneficial changes in cholesterol levels.

The ‘gut-brain axis’ is the term used to describe the communication between gut hormones, such as ghrelin and GLP-1, and the hypothalamus, particularly the melanocortin system. Ghrelin and GLP-1 are hormones released by the gut that have opposing actions on melanocortin circuits: ghrelin antagonizes melanocortin while GLP-1 is a melanocortin agonist. In the gut, GLP-1 increases insulin and decreases glucagon release. Ghrelin acts centrally to increase hunger, increase food intake, and enhance body mass. The melanocortin system is a potent regulator of the body’s energy status, affecting blood glucose and fat deposition. In this study, Perez-Tilva *et al*. showed that the melanocortin system plays a critical role in the regulation of cholesterol levels. First, the investigators injected the mice daily with ghrelin for one week. This produced mice with increased total body fat and plasma cholesterol, without affecting glucose or triglyceride levels. Next, they performed chronic intracerebroventricular (ICV) administration of either ghrelin or a melanocortin receptor antagonist (both of which functionally suppress melanocortin signaling in the hypothalamus), and found that cholesterol levels increased independence of food intake. Interestingly, this increase in cholesterol was driven primarily by increases in HDL cholesterol. When GLP-1 was injected ICV, a gut hormone thought to oppose the actions of ghrelin on melanocortin circuits, they found the opposite—reductions in HDL levels.

To see whether this modulation of HDL levels by melanocortin signaling is bidirectional (that is, whether HDL levels can be increased or decreased according to the activity of melanocortin circuits), the researchers either perfused the ventricles with a melanocortin agonist or antagonist. They found that enhancing melanocortin signaling resulted in lower HDL levels, while inhibiting melanocortin signaling resulted in higher HDL levels. These effects were independent of changes in body mass and did not change the characteristics of the HDL particles. Using radiolabeling of HDL particles, they found that increases in HDL levels after application of the melanocortin antagonist were due to changes in the reuptake of HDL particles (that is, less HDL was being cleared from the plasma). Therefore, brain melanocortin signaling appears to directly regulate cholesterol clearance from the plasma.

To determine the physiological role of melanocortin and ghrelin in cholesterol regulation, the authors produced modified animals that lacked ghrelin, the ghrelin receptor, or melanocortin receptors. Mutant animals that did not have ghrelin or its receptor had significantly lower cholesterol levels are being driven mainly by decreases in HDL. In contrast, mice lacking a specific melanocortin receptor, MC4R, had significantly higher cholesterol levels independent of gender or body mass. Hypothesizing that these effects were based on alterations in cholesterol reuptake, they measured levels of a particular receptor involved in HDL hepatic reuptake, Scarb1. In the mutants lacking ghrelin or ghrelin receptor, they found high levels of Scarb1, corresponding to an increase in cholesterol reuptake. To correlate this finding under more physiological conditions, they took normal mice and infused GLP-1 ICV. Similarly, they found increases in Scarb1 levels within the liver. This was expected, because GLP-1 antagonizes the effects of ghrelin *in vivo*. Thus, the investigators show that ghrelin signaling results in higher cholesterol and HDL levels by inhibiting hepatic reuptake. GLP-1 and melanocortin, on the other hand, encourage hepatic reuptake by increasing Scarb1 levels, resulting in lower cholesterol and HDL levels in the plasma. Although the investigators did find that modulation of melanocortin signaling resulted in changes in expression levels of genes responsible for hepatic cholesterol biosynthesis, this did not seem to be the dominant mechanism of cholesterol level regulation. Instead, the mechanism of cholesterol level regulation seemed to occur by regulation of Scarb1 expression and resultant hepatic reuptake.

In the last set of experiments, the researchers examined cholesterol levels in rats prone to diet-induced obesity (DIO). It has been shown that DIO animals tend to have a decrease in melanocortin signaling due to increases in melanocortin receptor antagonists. After maintaining these rats (and a cohort of obesity-resistant rats) on a high-fat diet for 10 months, the investigators found that the DIO rats were more obese, had higher cholesterol levels (including higher HDL cholesterol), and had less Scarb1 levels than controls. In rats switched from regular chow to a high-fat diet, ICV administration of melanocortin antagonists produced a similar result with higher HDL and total plasma cholesterol levels. When melanocortin signaling was enhanced by administering a melanocortin agonist, these effects were quickly reversed. Interestingly, the amount of melanocortin agonist required to reverse this effect was much lower in rats on a high-fat diet compared with those eating regular chow, suggesting hypersensitivity of the melanocortin receptors. The gut-brain axis has been and will continue to be an important area for obesity and metabolism research. One has to wonder if neurosurgical treatments for obesity and appetite disorders may involve selective modulation of the melanocortin circuits. Maybe using optogenetic approaches will shed some light on this.

This is a particularly interesting study in that it explores neural control of organ systems not usually thought of in the context of neurological dysfunction. Exploring the role of brain dysfunction or modulation in somatic disease may have an increasing role in the expansion of the neurosurgical field.

## SCREENING FOR NEW PRONEUROGENIC DRUGS PRODUCES PROMISING RESULTS[3]

### Key points

1. BrdU is a thymidine analog used to visualize nascent or proliferating cells. It incorporates into newly synthesized DNA, thus indirectly allowing researchers to see mitotically active cells or their derivatives.
2. P7C3 appears to be a safe, orally bioavailable compound that readily crosses the blood-brain barrier and results in greater BrdU-positive neurons in the subgranular zone (SGZ) of the dentate gyrus as well as decreased apoptosis. P7C3 also appears to protect against calcium-dependent mitochondrial toxicity.
3. P7C3 attenuates age-related learning and memory decline in aged animals, suggesting that: (1) cognitive decline among older animals is characterized by impaired SGZ neurogenesis or neuronal loss, and (2) P7C3 may enhance learning and memory abilities in animals suffering from neuronal loss and/or impaired neurogenesis in the hippocampus.

In this study, Pieper *et al*. performed a comprehensive molecular screen to look for new compounds that enhance hippocampal neurogenesis. Specifically, they screen the ability for these drugs to reverse the behavioral abnormalities and loss of neurogenesis seen in mice that have Npas3 knocked out (intentionally deleted by researchers), a gene thought to be responsible for adult neurogenesis. Approximately 1000 different chemicals were individually tested by perfusing mice ICV and also injecting them with BrdU, a thymidine analog that allows for visualization of proliferating or newly-born neurons. BrdU incorporates into newly synthesized DNA, thus indirectly allowing researchers to see mitotically active cells or their derivatives. Mice were then housed together and encouraged to exercise. Both of these activities have been found in research studies to enhance neurogenesis. The mice were then sacrificed and brain slices were examined for evidence of neurogenesis in the SGZ of the dentate gyrus. Initial screens resulted in the identification of a chemical named P7C3 that appeared to be proneurogenic. Furthermore, it is orally available and crosses the blood-brain barrier.

To see if P7C3 indeed enhances neuron formation in the hippocampus, the authors assayed P7C3-treated animals by looking at coexpression for other markers of neurogenesis, such as doublecortin, NeuN, and Prox-1. Although the BrdU-positive cells demonstrated neuronal markers, they did not stain for markers of astrocytes or oligodendroglia (proving that the newly formed cells with labeled BrdU were not glial cells but neurons). They then gave animals P7C3 for 7 days followed by a single pulse of BrdU intraperitoneally. They found that by 5 days after BrdU injection, the P7C3-treated animals had a 25% increase in the number of BrdU-positive cells in the SGZ compared with controls. By 30 days, the number of BrdU-positive cells in the treated group increased to 300% of controls!

At this point, the authors turned their attention towards the Npas3 knockout mice which have defects in adult neurogenesis. These mice also have abnormal dentate gyrus neurons, including attenuated dendritic branching and spine densities as well as hyperexcitability. To see if P7C3 could reverse or repair some of the deficits seen in the Npas3 mutant mice, pregnant female mice were given P7C3 throughout pregnancy and during lactation. P7C3 was continually administered to the pups through their third month of age. P7C3 administration corrected both the morphological and electrophysiological abnormalities of dentate gyrus neurons in the Npas3 mutant animals. Other markers of neurotransmission were also restored to normal levels by P7C3 therapy, including synapsin and synaptobrevin-2. Overall, the thickness of the dentate gyrus was increased in P7C3-animals and reduced apoptosis was seen. P7C3 was also found to promote the membrane integrity of mitochondria, reducing calcium-induced mitochondrial toxicity. No adverse effects of drug administration were noted.

Because Npas3 mutant mice cannot be assayed by standard behavioral tests to examine learning and memory, the authors decided to examine the effects of P7C3 administration to aged (18-month-old) rats. Like their previous experiments, P7C3 administration resulted in enhanced SGZ neurogenesis. Furthermore, rats treated with P7C3 fared better on learning and memory tasks. After sacrifice and examination of the brains, these animals had more SGZ neurons and reduced markers of apoptosis (programmed cell death). Interestingly, treated rats maintained their body weights better than controls, raising a question of P7C3’s role in age-related loss of body mass. Overall, this is an interesting study documenting the discovery of a compound that may lead to insights into mechanisms of neurogenesis and/or therapy for disorders characterized by impaired neurogenesis.

This experiment is notable for several reasons. First, it documents how researchers successfully performed a large-scale molecular screen to look for neuroactive chemicals. Second, these chemicals document a method of enhancing neurogenesis and reducing programmed cell death in the SGZ. Third, P7C3 appears to be protective against age-related cognitive decline and in genetic models of impaired neurogenesis. The obvious question that is not easily answered is how this applies to humans. Don’t be surprised if one day you see the P7C3 herbal supplement on store shelves next to Ginkgo biloba. Until then, we should take advantage of this chemical to learn more about the actual mechanisms underlying neurogenesis in the SGZ. This will be the key to developing advantaged strategies for neural repair in a wide array of cognitive disorders and dementias. It would be interesting to see how this fares in models of Alzheimer’s dementia.
